# Author response to: Recovery after breast cancer surgery following a recommendation of physical activity pre- and postoperatively (PhysSURG-B)—a randomized clinical trial

**DOI:** 10.1093/bjs/znab136

**Published:** 2021-05-05

**Authors:** J Heiman, E Haglind, R Olofsson Bagge

**Affiliations:** 1 Scandinavian Surgical Outcomes Research Group (SSORG), Department of Surgery, Institute of Clinical Sciences, Sahlgrenska Academy, University of Gothenburg, Gothenburg, Sweden; 2 Department of Surgery, Institute of Clinical Sciences, Sahlgrenska Academy, University of Gothenburg, Gothenburg, Sweden; 3 Sahlgrenska Breast Center, Department of Surgery, Sahlgrenska University Hospital, Gothenburg, Region Västra Götaland, Sweden; 4 Wallenberg Centre for Molecular and Translational Medicine, University of Gothenburg, Gothenburg, Sweden

*Dear Editor*

With great appreciation we read the comment by Dr Li *et al.* regarding the randomized PhysSurg-B trial^1^ of non-supervised physical activity before breast cancer surgery. 

Observational studies report that physically active patients have improved outcome, but no causal relationship can be determined. In our trial, an individualised intervention did not improve clinical or patient-reported outcomes of recovery. Neither did a similar intervention for colorectal cancer patients show differences in recovery or complications.

Risk factors for developing breast cancer was not studied in our trial, nor the effects on oncological outcome. Teras *et al*., referenced by Li *et al.*, show that women with sustained weight loss had lower risk of breast cancer. Selection bias and uncontrolled confounding influence these epidemiological data, acknowledged by the authors. Weight reduction is likely to lower breast cancer risk, but the question remains on how to accomplish this.

The DEDiCa study, also referenced by Dr Li *et al.*, randomized invited breast cancer patients within 12 months of diagnosis to two life-style programs, including diets, vitamin D and either no physical activity or brisk walks daily. Results have not yet been presented for disease-free survival. Benefits for quality of life are interesting but interpreted with caution, being a non-planned subgroup analysis without a control group.

To increase physical activity in a population is difficult, as for breast cancer patients. The PhysSurg-B trial intervention could be easily introduced as part of routine preoperative recommendations for all breast cancer patients, in contrast to many trials with supervised training in selected patients.

Recommending breast cancer patients physical activity is safe, but the level of evidence regarding how this can be accomplished and how this improves outcome is limited. We look forward to future studies addressing adherence to interventions and the safety of prehabilitation on delayed treatment.

*Disclosure*. The authors declare no conflicts of interest. 
